# A joint learning framework for multisite CBCT-to-CT translation using a hybrid CNN-transformer synthesizer and a registration network

**DOI:** 10.3389/fonc.2024.1440944

**Published:** 2024-08-08

**Authors:** Ying Hu, Mengjie Cheng, Hui Wei, Zhiwen Liang

**Affiliations:** ^1^ School of Mathematics and Statistics, Hubei University of Education, Wuhan, Hubei, China; ^2^ Bigdata Modeling and Intelligent Computing Research Institute, Hubei University of Education, Wuhan, Hubei, China; ^3^ Nutrition Department, Renmin Hospital of Wuhan University, Wuhan, China; ^4^ Department of Radiotherapy, Affiliated Hospital of Hebei Engineering University, Handan, China; ^5^ Cancer Center, Union Hospital, Tongji Medical College, Huazhong University of Science and Technology, Wuhan, China; ^6^ Hubei Key Laboratory of Precision Radiation Oncology, Wuhan, China

**Keywords:** CBCT, synthetic CT, deep learning, hybrid transformer, adaptive radiotherapy

## Abstract

**Background:**

Cone-beam computed tomography (CBCT) is a convenient method for adaptive radiation therapy (ART), but its application is often hindered by its image quality. We aim to develop a unified deep learning model that can consistently enhance the quality of CBCT images across various anatomical sites by generating synthetic CT (sCT) images.

**Methods:**

A dataset of paired CBCT and planning CT images from 135 cancer patients, including head and neck, chest and abdominal tumors, was collected. This dataset, with its rich anatomical diversity and scanning parameters, was carefully selected to ensure comprehensive model training. Due to the imperfect registration, the inherent challenge of local structural misalignment of paired dataset may lead to suboptimal model performance. To address this limitation, we propose SynREG, a supervised learning framework. SynREG integrates a hybrid CNN-transformer architecture designed for generating high-fidelity sCT images and a registration network designed to correct local structural misalignment dynamically during training. An independent test set of 23 additional patients was used to evaluate the image quality, and the results were compared with those of several benchmark models (pix2pix, cycleGAN and SwinIR). Furthermore, the performance of an autosegmentation application was also assessed.

**Results:**

The proposed model disentangled sCT generation from anatomical correction, leading to a more rational optimization process. As a result, the model effectively suppressed noise and artifacts in multisite applications, significantly enhancing CBCT image quality. Specifically, the mean absolute error (MAE) of SynREG was reduced to 16.81 ± 8.42 HU, whereas the structural similarity index (SSIM) increased to 94.34 ± 2.85%, representing improvements over the raw CBCT data, which had the MAE of 26.74 ± 10.11 HU and the SSIM of 89.73 ± 3.46%. The enhanced image quality was particularly beneficial for organs with low contrast resolution, significantly increasing the accuracy of automatic segmentation in these regions. Notably, for the brainstem, the mean Dice similarity coefficient (DSC) increased from 0.61 to 0.89, and the MDA decreased from 3.72 mm to 0.98 mm, indicating a substantial improvement in segmentation accuracy and precision.

**Conclusions:**

SynREG can effectively alleviate the differences in residual anatomy between paired datasets and enhance the quality of CBCT images.

## 1 Introduction

During radiotherapy, weight loss, tumor shrinkage and anatomical deformation may cause unwanted dose distribution and degrade the precision of dose delivery (1). Cone-beam computed tomography (CBCT) seems to be the most convenient way to obtain 3D anatomical information on the day of treatment. The role of recalculating the dose distribution and evaluating the necessity of replanning during CBCT is essential for adaptive radiation treatment (ART). However, cone beams generate a large amount of scatter in projection images, which results in severe artifacts, including cupping, shading, streaks, and inhomogeneities, hence reducing Hounsfield unit (HU) accuracy (2).

Many traditional methods have been introduced to improve the quality of CBCT images, including antiscatter accessories (3), scatter correction (4) and iterative reconstruction (5). In recent years, a commercial algorithm named Acuros CTS was proposed by Varian Medical Systems for clinical applications (6, 7). They corrected scatter by calculating primary and scatter images in the projection domain, followed by performing FDK-based reconstruction and statistical iterative reconstruction, and obtained clearer images and more accurate HU values. However, the direct use of CBCT in the adaptive pathway is still limited by the fact that the image quality of CBCT is considered significantly inferior to that of planning CT (pCT) in terms of the contrast-to-noise ratio and imaging artifacts (8, 9).

Recently, researchers have focused on improving the quality of CBCT images via convolutional neural networks (CNNs). Jiang et al. (10) proposed a deep residual CNN (DRCNN), which uses a residual U-Net framework, to learn the mapping function between scatter CBCT and scatter-free CBCT. Li et al. (11) utilized the DRCNN to convert CBCT images to synthetic CT (sCT) images for nasopharyngeal carcinoma (NPC) patients, maintaining the anatomical structure information of the CBCT images while correcting the HU distribution, similar to pCT images. Liang et al. (12) introduced a cycle-consistent generative adversarial network (cycleGAN) to generate sCT images for head and neck patients. Subsequently, cycleGANs have been used for patients with pelvic and/or prostate cancer (13, 14).One common deficiency of the abovementioned CNN-based models is their disregard of the global pixel relationships within images, which is primarily due to the limited receptive fields. These global relationships play crucial roles in achieving high-quality image restoration (15). To address this problem, the transformer architecture has recently been introduced to computer vision (16), offering the ability to model long-range dependencies and nonlocal information. Vision transformers (ViTs) (17) divide images into patches and employ multihead self-attention (SA) mechanisms to capture the relationships among patches. Chen et al. (18) obtained superior performance to that of a cycleGAN in the CBCT-to-CT translation task by using a transformer-based network. Nevertheless, the SA mechanisms of ViTs lead to quadratic computational complexity with respect to the image size, which poses challenges for low-level tasks that typically handle high-resolution images. Moreover, while ViTs excel in terms of capturing the global context and long-range dependencies, they may struggle to capture fine-grained local details and high-frequency components such as image edges. Additionally, a ViT typically requires larger datasets and a more extensive training process than other methods do for optimal generalization (19).

Another challenge is the local structural misalignment in paired datasets used for supervised learning. Rossi et al. (20) reported that the supervised learning approach can obtain better quantitative evaluation results but produces more blur and artifacts in qualitative evaluations, which is due to the higher sensitivity of the supervised training process to the pixelwise correspondence contained in the loss function. In practice, limited by the utilized scanning system or ethics, we usually cannot obtain paired images with perfect pixelwise matches from two modalities. To minimize the differences between paired images, previous studies (21, 22) have applied deformable image registration (DIR) to compensate for the anatomical mismatches resulting from patient position differences and potential internal anatomical changes. However, limited by the ability of DIR, the resulting datasets do not represent ideal pixelwise paired images and may introduce uncertainties in the training and evaluation processes of the constructed networks.

In this paper, we introduce SynREG to address the challenges encountered in sCT generation scenarios. Our approach combines a hybrid CNN-transformer synthesizer to capture both local and global information and a U-Net-based registration network to correct residual anatomy mismatches in the training pairs. By utilizing a supervised learning strategy, SynREG is trained on diverse anatomical datasets, which allows it to produce high-quality sCT images across multiple sites.

## 2 Materials and methods

### 2.1 Data collection and processing

Data from 135 patients with abdominal cancer, chest cancer or head and neck cancer were collected for training purposes in this study. Planning CT (pCT) and CBCT images were obtained from a CT simulator (Philips Medical Systems, Cleveland, OH, USA) and a kV CBCT system integrated on the Halcyon 2.0 system (Varian Medical Systems, Palo Alto, CA, USA), respectively. All CBCTs were scanned with a half-bowtie filter and reconstructed by the traditional filtered backprojection method with a 2-mm slice thickness, followed by our clinical scanning protocol. Detailed information about the scanning parameters is listed in **Table 1**. Deformable registration was implemented using MIM software (v.7.0.1, MIM Software Inc., Cleveland, OH, USA) to pair the pCT images with the CBCT images. The deformed CT volumes were resampled to the corresponding CBCT voxel spacing and then cropped to the CBCT dimensions and number of slices. Finally, a large dataset with 10,084 image pairs was used for training the model. In addition, data from an additional 23 patients with image pairs were collected for independent testing.

**Table 1:** CBCT scanning parameters used for head, thorax and pelvis patients.

CBCT Mode	Energy (kV)	Exposure (mAs)	CTDIvol (mGy)	DLP (mGy*cm)	Scan time (Sec)	Scan diameter (cm)
Head	100	126	3.33	49.9	16.6	28.2
Thorax	125	294	5.88	88.2	30.8	49.2
Pelvis	125	1080	21.6	324	36.7	49.2

### 2.2 SynREG framework

**Figure 1:**
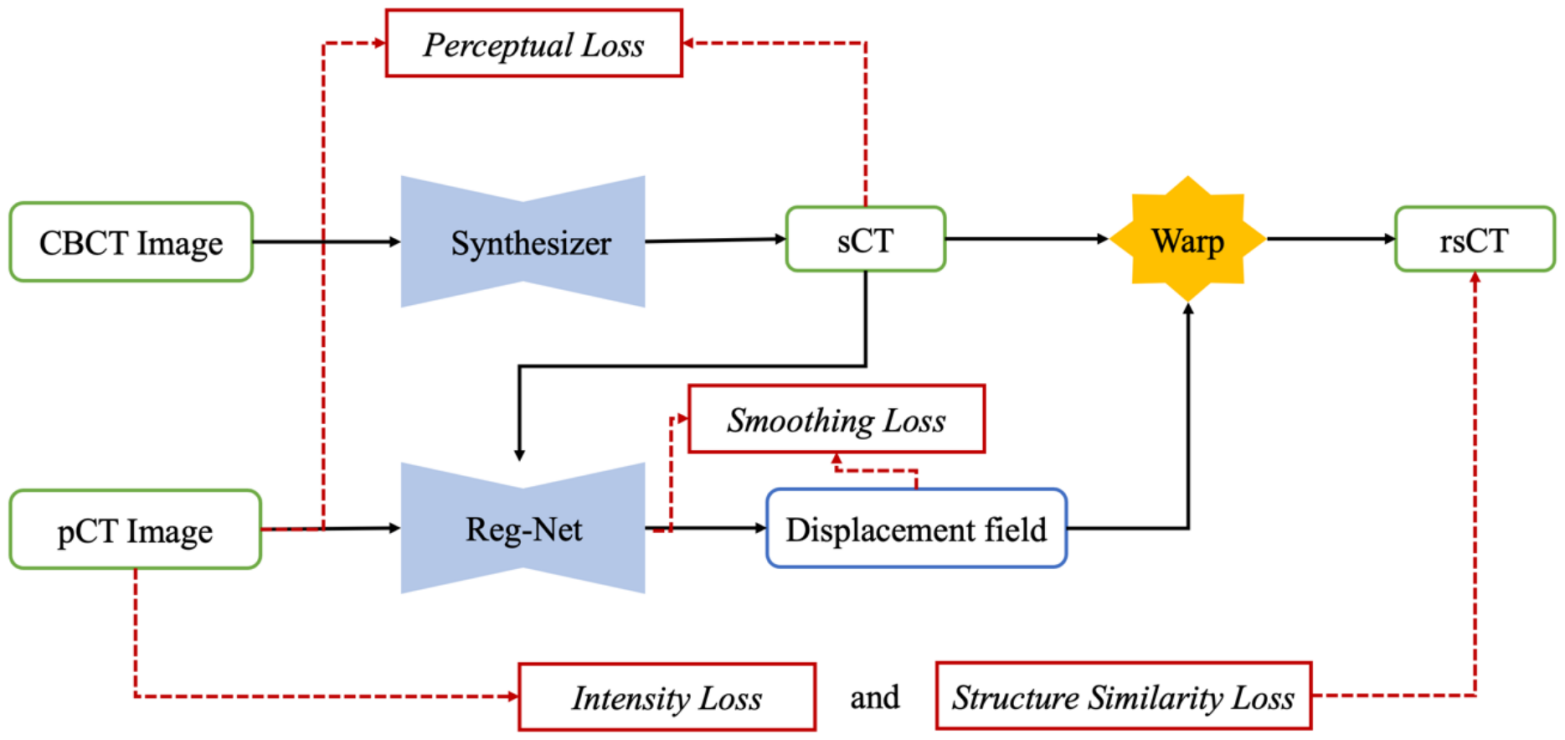
The overall framework of the proposed SynREG approach mainly includes a synthesizer and a registration network for achieving enhanced image quality and correcting residual anatomical mismatches.

We present the overall framework of our proposed SynREG algorithm in **Figure 1**. In our setup, each training sample consists of a pair of CBCT and pCT images, both with dimensions of 256x256. The CBCT image is initially passed through a synthesizer to generate an sCT image. Subsequently, a registration subnetwork (Reg-net) is employed to calculate the deformation vector field (DVF) between the sCT and pCT images. This allows for the manipulation of sCT to align with pCT. The synthesizer and Reg-Net are trained together using batches of paired CBCT and pCT images, ensuring optimal performance. In the following sections, we provide more detailed information on our model and the implemented loss functions.

#### 2.2.1 Hybrid CNN-transformer synthesizer

**Figure 2:**
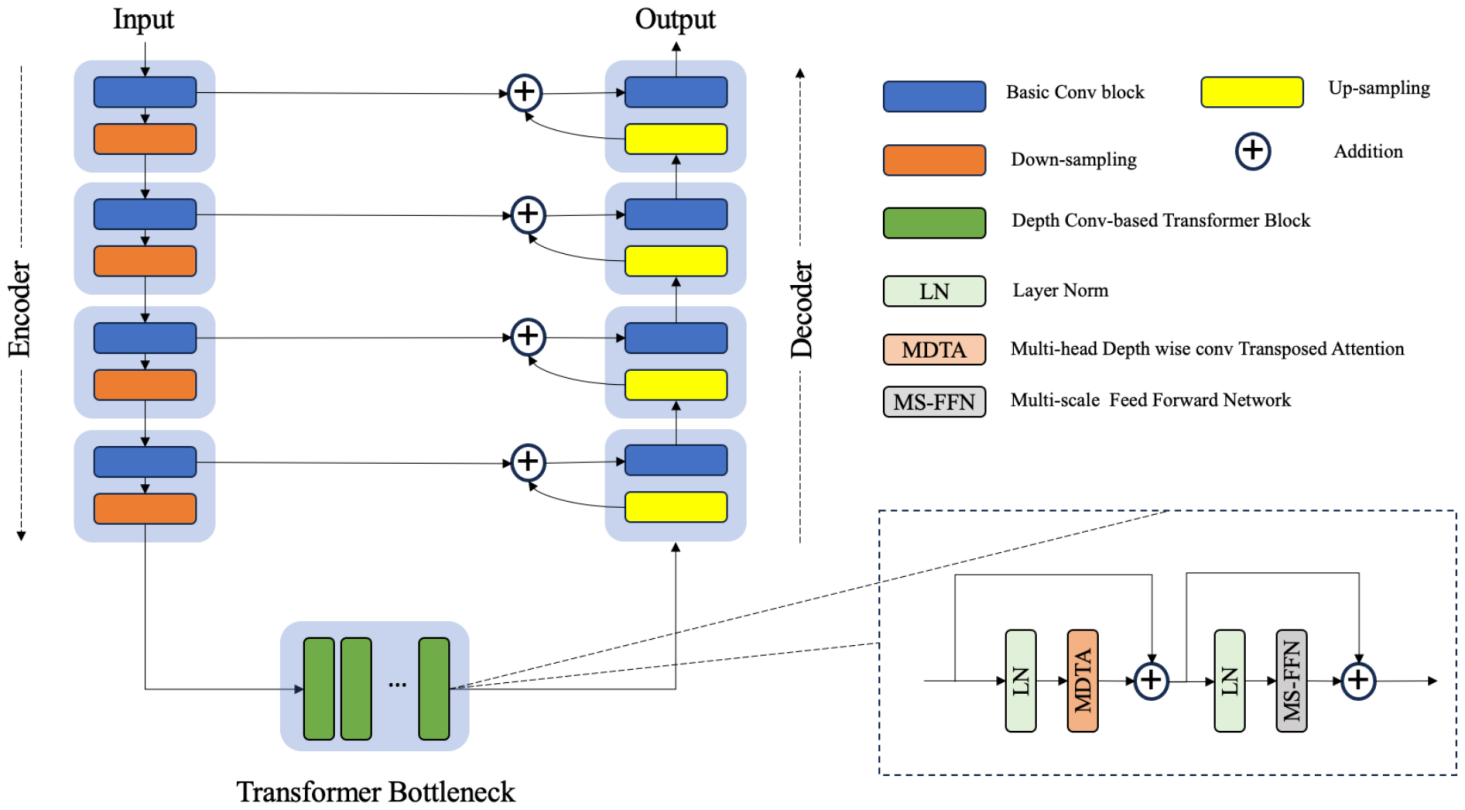
The architecture of the hybrid CNN-transformer synthesizer. The synthesizer was constructed on the basis of a U-net structure composed of an encoder, a transformer bottleneck and a decoder. Each transformer block contains a multihead depthwise convolution transposed attention module (MDTA) and a multiscale feed forward network module (MS-FFN).

Due to similar physical processes, CBCT can be viewed as a potentially degraded version of a CT image. Hence, choosing the most critical features while eliminating undesirable features in the channel dimension is crucial for noise suppression and artifact removal. Inspired by Restormer (23), we employ SA across the feature dimension instead of the spatial dimension to construct the fundamental transformer block. Consequently, we introduce a hybrid CNN-transformer synthesizer that incorporates a stack of nine depth convolution-based transformer blocks (DTBs) organized in a UNet architecture (24) (**Figure 2**).

Given a CBCT image $I_{CBCT}\in {\mathbb{R}}^{H\times W\times 1}$ , the synthesizer first applies a 1 × 1 convolution to obtain low-level feature maps $F_{0}\in {\mathbb{R}}^{H\times W\times 32}$ , where H×W represents the spatial resolution. Subsequently, the encoding path of UNet extracts these shallow features $F_{0}$ through four consecutive layers of convolution and downsampling. The features extracted at each layer are relayed to the corresponding layers of the decoding path via skip connections, whereas the bottom-level features are passed to the stack of DTBs. With this design, the skip connections effectively facilitate the high-frequency features to the decoder, whereas the DTB bottleneck serves as an effective approach for learning pairwise relationships among low-frequency features.

**Figure 3:**
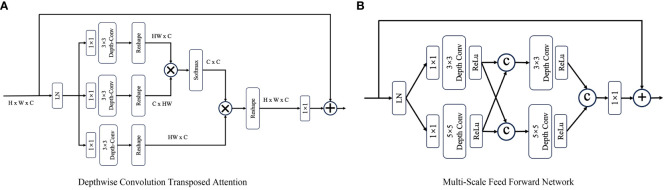
The architecture of **(A)** the depthwise convolution transposed attention (DTA) module and **(B)** the multiscale feed forward modules (MS-FFN). Where 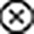 and © refer to the multiplication and concatenation operations, respectively.


A DTB consists of two fundamental components: a multihead depthwise convolution-transposed attention (MDTA) module and a multiscale feedforward network (MSFN), as shown in **Figure 3**. Within the architecture, the MDTA module applies SA across channels to compute the cross covariance across the channels and generate an attention map that implicitly encodes global context information. This attention map is then used to weight the feature maps, allowing the model to focus on the most relevant information. **Figure 3A** illustrates the architecture of a single-head DTA, which initially encodes channelwise context through 1 × 1 convolutions, followed by 3 × 3 depthwise convolutions to capture spatial local context within each channel. The MDTA extends this foundation by utilizing multiple parallel heads, each of which independently focuses on distinct parts of the input. Then, SA across the channels is applied to generate attention. MDTA has linear complexity, hence reducing the temporal and memory complexity of the network. The attention mechanism is generally formulated as Equation (1).

(1) $$Att\left(Q,K,V\right)=softmax\left(\frac{QK^{T}}{\lambda }\right)V$$

where λ is a learnable scaling parameter that controls the magnitude of the dot product of Q and K .

The MSFN module (**Figure 3B**) is applied after the MDTA module, and its effectiveness has been verified by Chen et al. (25). It consists of two multiscale local information extraction operations. After performing layer normalization, a 1x1 convolution is applied to expand the channel dimensionality. Then, the expanded features are fed into two parallel branches, in which 3x3 and 5x5 depthwise convolutions are employed to enhance the multiscale local information extraction process. The extracted features derived from both branches are subsequently concatenated. After another multiscale information extraction operation, a 1x1 convolution is used to keep the size of the output tensor matched with that of the tensor that was initially fed into the MSFN.

#### 2.2.2 Registration network

The Reg-Net employed in this study is based on the work of Kong et al. (26). Its objective is to acquire prior knowledge about the DVF from the input sCT and pCT images. The DVF represents the displacement of each pixel, and by warping the sCT using the calculated DVF, the resulting registered sCT (referred to as rsCT) can be optimized to minimize its differences from the pCT via the pixelwise intensity loss function.

Reg-Net is a modified version of the U-Net architecture that consists of seven downsampling blocks, three residual blocks, and seven upsampling blocks. In each downsampling block, features are extracted at various levels with different numbers of filters, namely, 32, 64, 64, 64, 64, 64, 64 and 64. The upsampling process is the counterpart of the downsampling process and incorporates skip connections to collect the corresponding blocks at each level. Finally, Reg-Net outputs DVFs across the horizontal and vertical dimensions, ensuring accurate reconstruction of a high-resolution DVF representation.

#### 2.2.3 Loss functions

The synthesizer employs a perceptual loss for computing the feature similarity between sCT and pCT images at multiple levels. To extract deep multilevel features and structural information, we introduce the deep image structure and texture similarity (DISTS) index as the perceptual loss because it unifies texture similarity and structural similarity into a single index. The loss function is formulated as Equation (2).


(2) $${ℒ}_{perceptual}=D\left(x,y;\alpha ,\beta \right)=1-\;\sum_{i=0}^{m}\sum_{j=1}^{n_{i}}\left(\alpha_{ij}l\left({\tilde{x}}_{j}^{\left(i\right)},{\tilde{y}}_{j}^{\left(i\right)}\right)+\beta_{ij}k\left({\tilde{x}}_{j}^{\left(i\right)},{\tilde{y}}_{j}^{\left(i\right)}\right)\right)$$

where x and y represent the sCT and pCT images, respectively. i represents the convolution layers, and j represents the channel in the i th convolution layer. $\alpha_{ij}$ and $\beta_{ij}$ are positive weights, which are pretrained via a variant of the visual geometry group (VGG) network. l(·) and k(·) are the defined texture similarity and structure similarity, respectively. The details of the DISTS index were described by Ding et al. in 2020 (27).

Reg-Net has three loss functions, including intensity loss, structural similarity loss and smoothing loss. Here, we use the Charbonnier loss (28) as the intensity loss, which compares the intensity difference between the rsCT and pCT images (referred to as x and y , respectively) and is formulated as Equation (3).


(3) $${ℒ}_{intensity}=\sqrt{\text{||}y-x{\text{||}}^{2}+e^{2}}$$

where e is a constant that is set to $10^{-3}$ .

Structural similarity is measured using locally normalized cross-correlation (LNCC) (29), which emphasizes the anatomical similarity between rsCT and pCT images, and defined as Equation (4).


(4) $${ℒ}_{structure}=\frac{1}{N-1}\sum_{i=1}^{N}\frac{\left(x_{i}-\mu_{x_{i}}\right)\left(y_{i}-\mu_{y_{i}}\right)}{\sigma_{x_{i}}\sigma_{y_{i}}}$$

where N is the number of samples and where $\left(\mu_{x_{i}},\;\mu_{y_{i}}\right)$ and $\;\left(\sigma_{x_{i}},\;\sigma_{y_{i}}\right)$ denote the means and standard deviations of $x_{i}$ and $y_{i}$ , respectively.

The smoothing loss is defined in Equation 5 to evaluate the smoothness of the deformation field and minimize its gradient.


(5) $${ℒ}_{smooth}=E_{x,\;y}\left[\|\nabla R\left(x,\;y\right){\|}^{2}\right]$$

The total loss of the proposed SynREG approach is Equation (6).


(6) $$ℒ={ℒ}_{perceptual}+\lambda_{1}{ℒ}_{intensity}+\lambda_{2}{ℒ}_{structure}+\lambda_{3}{ℒ}_{smooth}$$

## 3 Experiments

### 3.1 Implementation details

The CBCT/CT image pairs obtained from 136 patients were randomly divided into a training set and a validation set at a ratio of 0.9 to 0.1. The training set comprised 9,076 pairs of images, whereas the validation set consisted of 908 pairs. Both the CBCT and CT images had an HU value threshold range set to [-1000, 2200], with any values outside this range being set to the nearest threshold values. The HU values were subsequently normalized and mapped to the range of (-1, 1). During the training process, a patch with 256x256 dimensions was randomly cropped from each processed image and used as a network input. Additionally, data augmentation techniques such as random flipping and rotation were applied with a probability of 0.3.

The adaptive moment estimation (Adam) optimizer was employed for optimization with the momentum parameters set to β1 = 0.5 and β2 = 0.999 .  A superconvergence cosine annealing strategy with a warm-up learning rate was implemented during training (30). Initially, the learning rate was set to 0.0001, and it gradually increased to a maximum of 0.1 at epoch 50. Then, it gradually decreases to zero by epoch 200 following a cosine function.

During the training process, $\lambda_{1}$ , $\lambda_{2}$ and $\lambda_{3}$ in the loss function were empirically set to 5, 1 and 1, respectively. The intensity loss between the pCT and rsCT images was calculated for the validation data every 10 epochs. The model that achieved the minimum intensity loss was saved as the best model.

### 3.2 Image quality evaluation metrics

To quantitatively evaluate the image quality of the images generated by each model in comparison with the reference pCT images, we employed commonly used metrics such as the mean absolute error (MAE), root mean square error (RMSE), peak signal-to-noise ratio (PSNR), and structural similarity index measure (SSIM). These metrics are defined by Equations (7)–(10).


(7) $$MAE\left(I_{1},I_{2}\right)=\;\frac{1}{n_{i}n_{j}}\sum_{x,y}^{n_{i}n_{j}}\left|I_{1}\left(x,y\right)-I_{2}\left(x,y\right)\right|$$

(8) $$RMSE\left(I_{1},I_{2}\right)=\;\sqrt{\frac{1}{n_{i}n_{j}}\sum_{x,y}^{n_{i}n_{j}}{\left|I_{1}\left(x,y\right)-I_{2}\left(x,y\right)\right|}^{2}}$$

(9) $$PSNR\left(I_{1},I_{2}\right)\;=\;10\times log_{10}\left(\frac{P^{2}}{RMSE{\left(I_{1},\;I_{2}\right)}^{2}}\right)$$

(10) $$SSIM\left(I_{1},I_{2}\right)\;=\;\frac{\left(2\mu_{I_{1}}\mu_{I_{2}}+c_{1}\right)\left(2\sigma_{I_{1},I_{2}}+c_{2}\right)}{\left(\mu_{I_{1}}^{2}+\mu_{I_{2}}^{2}+c_{1}\right)\left(\sigma_{I_{1}}^{2}+\sigma_{I_{2}}^{2}+c_{2}\right)}$$

where $I_{1}$ and $I_{2}\;$ represent two different images used for comparison purposes; I(x,y) is the HU value of pixel (x,y) in image I ; ${\text{n}}_{\text{i}}{\text{n}}_{\text{j}}$ is the total number of pixels in image I ; P is the maximum HU range of the image; and μ σ , $c_{1}$ and $c_{2}$ are the same as those defined above.

### 3.3 Segmentation evaluation

Automatic segmentation is an important aspect of clinical work that can improve the efficiency of the ART workflow. In this study, a commercial AI-based autocontour module of UIH TPS (v.1.0, United Imaging Healthcare Co., Shanghai, China) and a well-known open-source tool, TotalSegmentator (TS) (31), whose accuracy and robustness have been tested on diverse datasets, were adopted to evaluate the segmentation results. Considering the limited field of view (FOV) of CBCT, we selected the brainstem and parotids from the head cases and the bladder and rectum from the pelvis cases for testing. We generated automatic segmentations on the pCT, CBCT and sCT images. The segmented pCT contours were regarded as the ground truths, and the contours from the other image modalities were compared. The Dice similarity coefficient (DSC) and mean distance to agreement (MDA) were used to evaluate the segmentation accuracy. A higher DSC and lower MDA indicate better consistency between the segmented contours and the ground truths.

### 3.4 Statistical analyses

To determine if the data from the two groups were significantly different, we adopted the paired t test if the data were normally distributed; otherwise, the Wilcoxon signed-rank test, a nonparametric test for paired samples, was adopted. A statistical significance level of p < 0.05 was used.

## 4 Results

### 4.1 Comparison with other benchmark models

**Figure 4:**
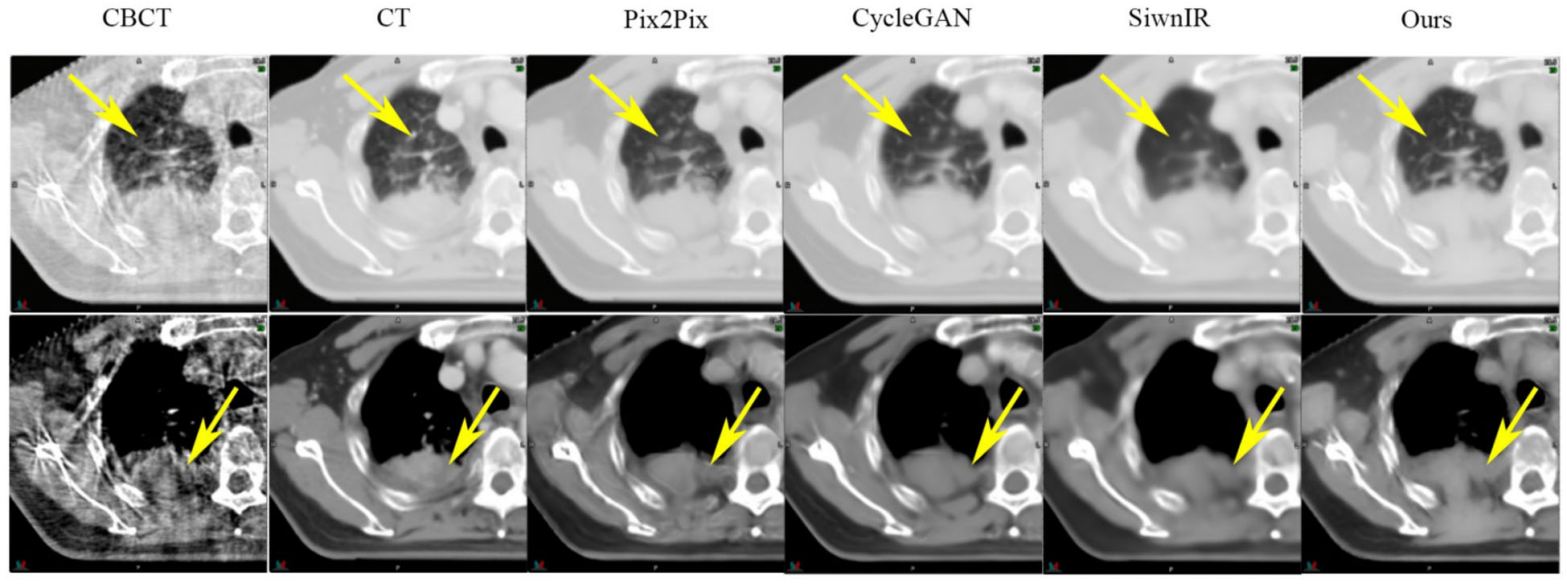
Comparison of the sCT images generated by the SynREG model and other benchmark models. The yellow arrows highlight the areas with apparent visual differences. The upper row shows an example slice in the lung window/level, whereas the lower row shows the same slice in the soft tissue window/level.

SynREG was compared with three other image benchmark models: pix2pix (32), cycleGAN (33) and SwinIR (34). The results demonstrate that our method outperforms these benchmarks, exhibiting a remarkable ability to generate high-quality sCT images that capture intricate textures and faithfully preserve anatomical structures. As evidenced by the yellow arrows in **Figure 4**, the sCT images generated by SynREG show the detailed texture of the bronchi and the precise shape of the tumor, both of which are crucial for accurate clinical diagnosis and tumor delineation.

**Figure 5:**
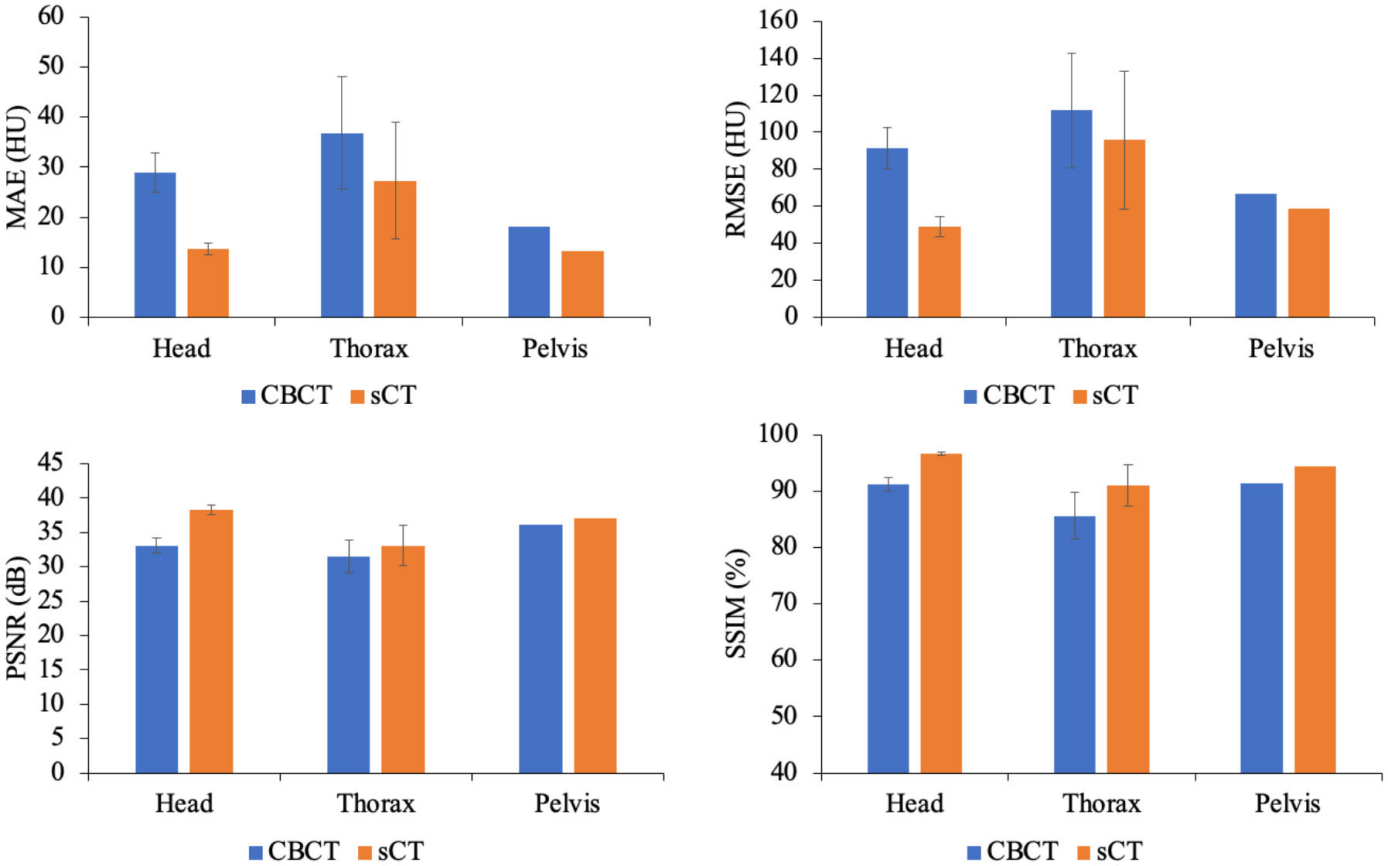
Comparison of the MAEs, RMSEs, PSNRs and SSIMs produced for the CBCT and sCT images at individual sites, including the head, thorax and pelvis, in the test dataset.

**Table 2** presents the quantitative results on the test dataset, revealing significant improvements in both the MAE and SSIM, with p values less than 0.01. Furthermore, **Figure 5** illustrates the performance metrics for individual sites, demonstrating substantial reductions in the MAE and RMSE, along with notable increases in the SSIM for all sites. This finding reveals the generalizability of our model.

**Table 2:** Quantitative comparison of the test dataset among different benchmark models.

	MAE (HU)	RMSE (HU)	PSNR (dB)	SSIM (%)
CBCT	26.74 ± 10.11	87.17 ± 27.43	33.86 ± 2.79	89.73 ± 3.46
Pix2Pix	18.17 ± 8.29	64.45 ± 27.23	35.53 ± 3.07	91.97 ± 2.89
CycleGAN	18.32 ± 8.66	66.84 ± 27.64	36.18 ± 3.06	93.60 ± 2.97
SwinIR	17.97 ± 8.52	66.49 ± 28.31	36.40 ± 3.18	93.65 ± 2.98
Ours	16.81 ± 8.42	64.10 ± 27.82	36.59 ± 3.12	94.34 ± 2.85

The reported values are the average ± STD results.

**Figure 6:**
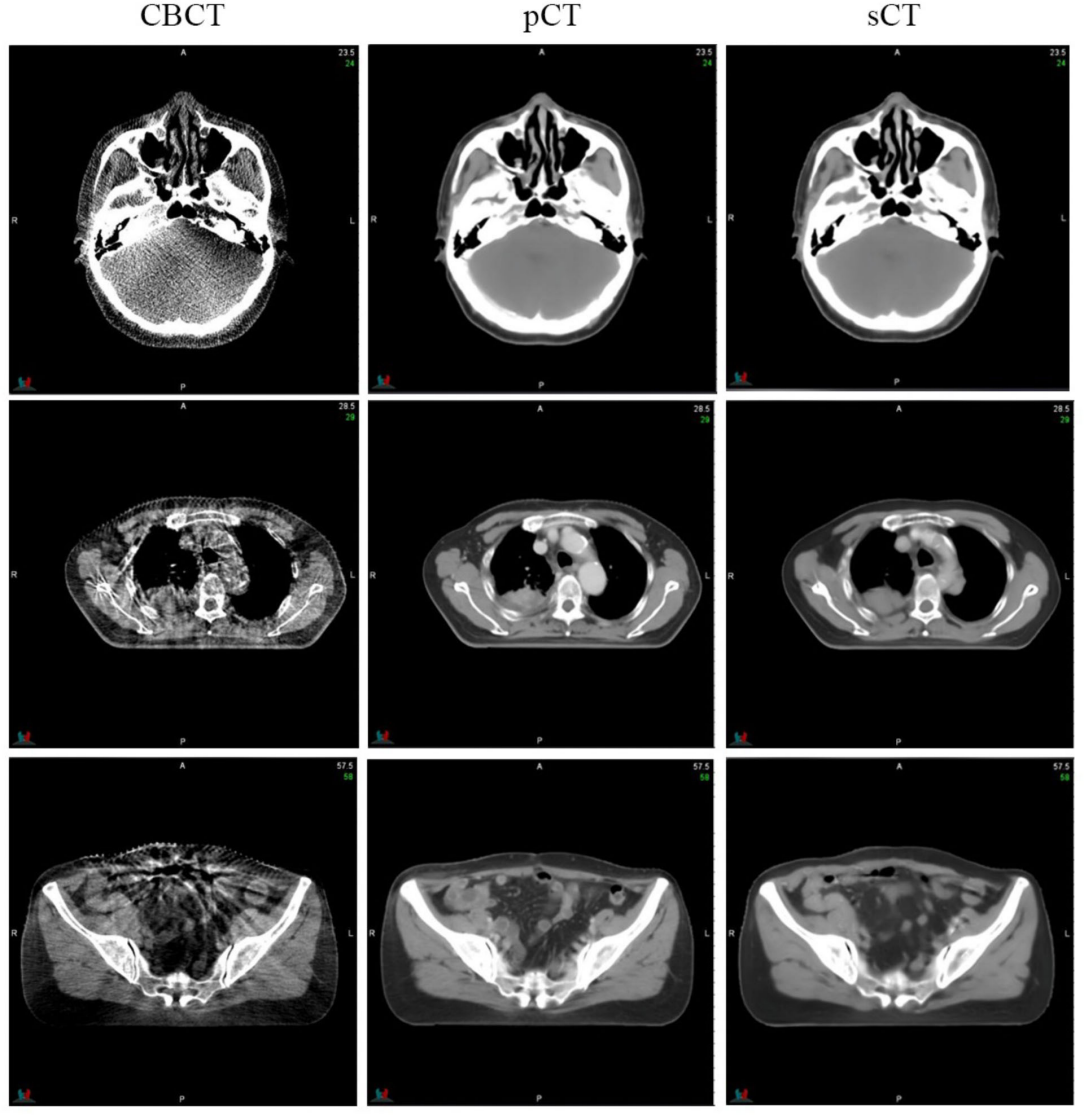
Examples of image slices obtained for the head, thorax and pelvis cases. The display window ranged from -400 HU to 400 HU.

**Figure 6** highlights the visual improvements achieved by SynREG for multisite cases. The original CBCT image exhibits severe noise, spatial nonuniformity and various artifacts, including beam hardening artifacts and streak artifacts. However, the sCT images generated by our method exhibit remarkable visual performance, effectively reducing noise and eliminating artifacts. This finding demonstrates the robustness and effectiveness of our proposed method in generating clinically relevant sCT images.

### 4.2 Ablation experiments

To investigate the impact of Reg-Net and varying loss combinations on model performance, we evaluated four distinct configurations: M1, M2, M3 and M4. M1 serves as a baseline, employing only the synthesizer subnetwork and the L1 loss; M2 incorporates the SynReg architecture in conjunction with the L1 loss; M3 incorporates the SynReg with perceptual loss; and M4, our proposed method, leverages both perceptual loss and L1 loss within the SynReg framework. The quantitative results from these experiments are presented in **Table 3**. Reg-Net contributes to improving the MAE and SSIM of the model by mitigating local structural misalignments. The perceptual loss enhances the PSNR and SSIM by generating high-fidelity images, though it may not directly contribute significantly to reducing the MAE. Our proposed method, M4, integrates all of these components effectively, achieving superior results.

**Table 3:** Quantitative results of the ablation experiment.

	MAE (HU)	PSNR (dB)	SSIM (%)
M1 (Syn Only + L1)	18.54 ± 8.81	34.55 ± 3.06	91.80 ± 2.77
M2 (SynReg + L1)	17.48 ± 8.09	35.87 ± 2.93	93.96 ± 2.84
M3 (SynReg + Lperceptual)	17.89 ± 8.44	36.32 ± 3.27	94.38 ± 2.96
M4 (SynReg + Lperceptual + L1)	16.81 ± 8.42	36.59 ± 3.12	94.34 ± 2.85

The compared models (M1–M4) are trained with different settings.

### 4.3 Training on an individual dataset versus the entire dataset

When focusing on the head and neck dataset alone, our model trained on this dataset achieved a mean MAE of 14.18 HU, significantly reducing the intensity error for those cases. However, when applied to the thorax and pelvis cases, no MAE reduction was observed, with values of 38.84 HU and 28.14 HU, respectively. This highlights the model’s limited generalizability when trained on a single dataset. Conversely, the model trained on the entire dataset (synREG) consistently improved the MAE across anatomical sites. Notably, it achieved a lower MAE of 13.67 HU for head and neck cancer patients, emphasizing the importance of diverse and representative data for robust, generalizable models.

### 4.4 HU calibration

**Figure 7:**
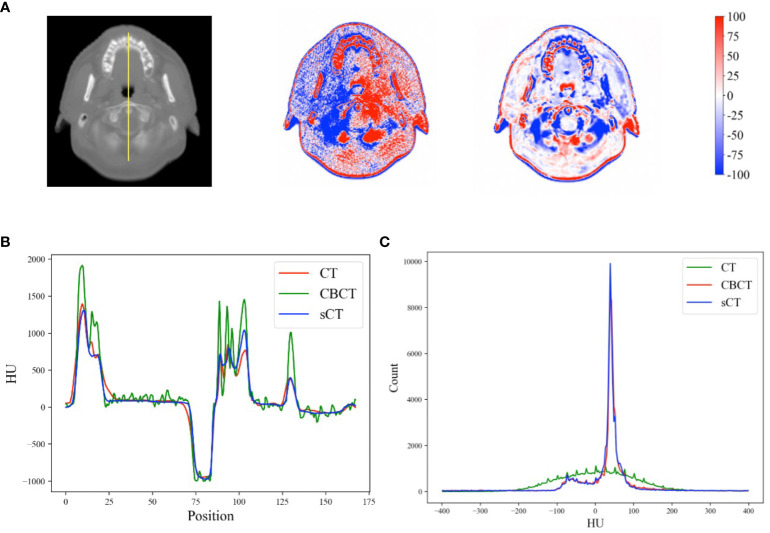
**(A)** HU differences between the CBCT, sCT and rsCT images and the pCT image for a head case example. **(B)** HU profiles of the yellow line in **(A)**. **(C)** HU distributions within the range of [-400, 400] for the example cases across all image modalities.

**Figure 7A** shows the HU calibration performance. By referring to the pCT image as the reference image, the HU difference relative to the sCT image was significantly improved. In the high-frequency areas (i.e., edges) of the sCT image, the HU differences were greater than those in other areas, indicating intrinsic anatomical differences. The HU profiles of the yellow line in **Figure 7A** obtained across bone, soft tissue and air are shown in **Figure 7B**. Furthermore, the HU distributions of the example case are shown in **Figure 7C**. Our model effectively mapped the intensity distribution of the CBCT image to the pCT image.

### 4.5 ROI contouring

The DSC and MDA values obtained for 15 patients, including 8 head patients and 7 pelvis patients, are presented in **Tables 4**, **5**, respectively. Thorax cases were excluded because of the limited FOV of the CBCT scanning system, preventing full organ scanning.

**Table 4:** Comparison of the mean Dice coefficients for automatic segmentation of various organs between CBCT and sCT images.

		Brain Stem		Parotide_L		Parotide_R		Bladder		Rectum	
		CBCT	sCT	CBCT	sCT	CBCT	sCT	CBCT	sCT	CBCT	sCT
Dice	UIH	0.63	0.89	0.89	0.91	0.87	0.90	0.63	0.70	0.68	0.68
	TS	0.59	0.89	0.88	0.91	0.88	0.91	0.60	0.70	0.68	0.69
	Mean	0.61	0.89	0.88	0.91	0.88	0.91	0.62	0.70	0.68	0.68

**Table 5:** Comparison of the mean distance to agreement (MDA) for automatic segmentation of various organs between CBCT and sCT images. (mm).

		Brain Stem		Parotide_L		Parotide_R		Bladder		Rectum	
		CBCT	sCT	CBCT	sCT	CBCT	sCT	CBCT	sCT	CBCT	sCT
MDA	UIH	3.51	0.98	1.00	0.77	0.98	0.79	3.45	3.41	3.60	3.07
	TS	3.93	0.98	1.05	0.77	0.98	0.79	3.53	3.42	3.66	3.05
	Mean	3.72	0.98	1.03	0.77	0.98	0.79	3.49	3.42	3.63	3.06

**Table 4** presents the DSC outcomes achieved with the UIH and TS tools, revealing a consistent enhancement in segmentation accuracy across most regions when sCT images were used instead of CBCT. Notably, the brainstem mean DSC significantly improved from 0.61 to 0.89. Additionally, **Table 5** shows that the mean MDA values of the brainstem significantly decreased from 3.72 mm when CBCT was used to 0.98 mm when sCT was used. For the parotid glands, we also observed positive trends, highlighting the role of sCT in improving contour accuracy through image quality enhancement. Although the DSC and MDA values for the bladder and rectum do not significantly change, sCT images still provide slightly higher segmentation accuracy in these regions.

## 5 Discussion

In this study, we employed a deep learning approach to translate multisite CBCT images to sCT images. To utilize paired data with local structural misalignment for training, we proposed SynREG, which disentangled the sCT generation and anatomical correction processes via a synthesizer and a Reg-Net, respectively. With this approach, we can train the model in a supervised manner, which has demonstrated the advantages of efficiency in data and computation due to its explicit learning objective (35). Moreover, the transfer learning ability of a supervised pretraining model can be further enhanced when models are trained on increasingly expansive datasets (36). In this study, we trained a singular model capable of generating sCT images for the head, thorax and pelvis. **Figures 4**, **5** demonstrate the efficacy of our proposed model in enhancing image quality across all sites through quantitative and qualitative evaluations. The quantitative results (**Table 2**) indicate that SynREG outperforms the other unsupervised benchmark models and outperforms the model trained solely on the head and neck dataset.

Although CBCT and pCT images share anatomical similarities, simple HU mapping is not sufficient for generating sCT images because noise and artifacts may easily introduce nonlinearity in the intensity profile mapping process. To capture more complete local and global relationships for high-quality image restoration and generation. We introduced a hybrid CNN-transformer model to enhance the representation ability of the model while saving computer resources. Traditional ViTs require many computational resources and large datasets. Various self-attention computation methods, such as local window attention (37, 38), channel dimension attention (39), and sparse self-attention (40), have been proposed to reduce model complexity. SwinIR (34) utilizes a swin transformer to perform image restoration. Compared with vanilla ViT, the swin transformer employs a shifted window mechanism to combine local attention with global attention, enabling the capture of global context information while maintaining computational efficiency. Despite its strengths, the patch embedding utilized in the Swin transformer encounters inherent limitations in capturing local details, particularly for the preservation of fine details (41), as shown in **Figure 4**. Hence, we did not opt for patch embedding and instead adopted a depthwise convolutional transposed attention mechanism, which proves to be more effective for enhancing the representation of fine details and for generating high-quality images. Additionally, this approach offers the advantage of linear computational complexity, making it a more efficient solution for our task.

The segmentation of target structures and organs at risk is a crucial component of the radiotherapy workflow. Most deep learning-based autosegmentation models applied in radiotherapy are trained with CT and/or MR images, significantly enhancing the efficiency and accuracy of the task (42). However, owing to the limited generalization ability of autosegmentation models, it is not advisable to train a model on one image modality and directly apply it to another modality, as this often leads to suboptimal performance.

Our results demonstrate that the DSC of CBCT is the lowest. This can be attributed to data distribution differences caused by different image modalities (**Figure 7C**), as well as the inferior quality of CBCT images. Following the conversion of CBCT to sCT images, both the data distribution disparity and image quality were enhanced, resulting in an increase in the DSC value. Notably, for the brainstem, which has low contrast and is overwhelmed by noise in CBCT images, the increase in DSC was primarily due to the essential image quality enhancement process. On the other hand, for the bladder and rectum, which have higher contrast and can be easily discriminated in CBCT images, the relatively lower DSC was primarily due to the structural mismatches caused by variations in bladder and rectum fullness. Therefore, the increase in DSC yielded by sCT was not significant for these regions. Similarly, the MDA results also supports these findings.

**Figure 8:**
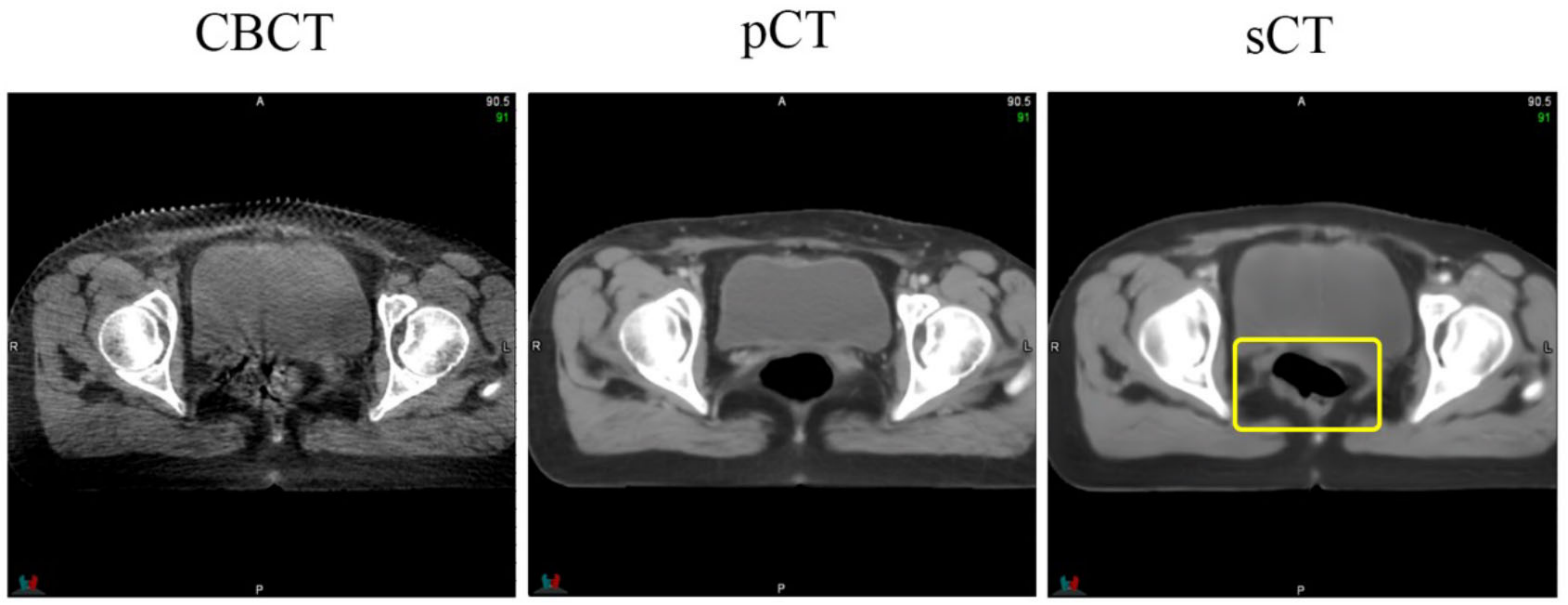
An instance of hallucinations on an sCT image. The area highlighted by the yellow box indicates the location of the hallucinations, which are not present in the CBCT image.

Supervised learning often relies on high-quality datasets for optimal model performance. Our method mitigates the need for precisely matched paired images by disentangling image generation from anatomical correction. This approach is capable of handling most scenarios and generating high-quality sCT images. However, we also encountered unreliable results in certain scenarios, especially for cavities. As shown in **Figure 8**, while the bladder structure of the CBCT image was effectively preserved, the appearance of a cavity on the sCT image was unreliable. This is because performing image registration in cases with large deformations remains a significant challenge, thereby compromising dataset quality. Exploring ways to enhance dataset quality and/or incorporating a more targeted loss function are potential approaches to address this limitation and achieve a more accurate clinical implementation model.

In this study, we used a joint learning framework called SynREG to address the challenge of training a model with imperfectly aligned CBCT−CT paired data. Our approach involved proposing a hybrid CNN-transformation model for sCT generation and a registration network for anatomical correction. Additionally, we explored the feasibility of training a singular model for generating multisite sCT images. Our quantitative and qualitative findings demonstrated the superior performance of our method and its potential application in ART.

## Data availability statement

The raw data supporting the conclusions of this article will be made available by the authors, without undue reservation.

## Author contributions

YH: Methodology, Writing – original draft. MC: Data curation, Formal analysis, Writing – original draft, Writing – review & editing. HW: Data curation, Writing – original draft. ZL: Conceptualization, Supervision, Writing – review & editing.
